# Revelations by Electricity

**Published:** 1886-01

**Authors:** F. T. Paine

**Affiliations:** Comanche, Texas


### REVELATIONS BY ELECTRICITY IN RHEUMATISM AND NEURALGIA.

*By F. T. Paine, M. D., Comanche, Texas.*

For Daniel's Texas Medical Journal.

IN July, 1885, Mr. J. E. DeWitt, aged fifty-eight, presented himself at my clinic, led by a pilot, he being blind from, as he claimed, Neuralgia, and from which he had frequently suffered, and lost his right eye. On examination, I found the left eye intensely congested ; in fact, I could scarcely discover anything but a mass resembling coagulum filling the orbit. He had slept none, he said, for six nights and days, and was suffering intensely. A thirty-minutes seance with the electric current relieved the patient so much that he left for the night. On the next day he could begin to distinguish day from night, and, after the third seance, dismissed his pilot and could sleep soundly all night.

On one occasion, while applying the electric current directly to the globe, he detected, as he said, a spot about the center of the eyes that had no feeling whatever, or upon which the Faradic current produced no impression. Upon this spot, as near as possible, for I had to rely upon his feeling entirely, I concentrated the current until he felt it. He instantly exclaimed, "I can feel it now, and can see better, and the current is pleasant.'' From this time on he continued to improve, and in one week from the time he commenced treatment, he was enjoying his usual health, with as good sight as he has had for years.

Another discovery in this case, was the fact that, by electricity alone, the fever and inflammation were so effectually removed that the patient became alarmed lest his eyelids were frozen, as they felt so cold.

The next case was that of Rev. P. W. Graves, who presented himself in August, for chronic sore eyes, with granulated lids and ulcerated cornea, and numerous nebulæ. He, too, being entirely blind, was led by a pilot. The Farradic current was used through an eye cup of water. His first exclamation was, ''Doctor, you have forgotten to turn on the current; I don't feel anything.'' But finding all right, I continued the application until he began to feel the electricity darting into the eye back and forth, until presently he exclaimed, ''I feel it all over the eye now, and can see light.'' The feeling, he said, was delightful. He was led home, and slept as he had not slept for months, and without the least attention to his eyes during the night. He attended the clinic regularly, and would frequently say that his eyes felt like they had lumps of ice upon them.

The inflammation has entirely subsided, the granulations and ulcerations have disappeared, and he has reported to his Conference for regular work as an itinerant minister.

The point I wish to present in these two cases is the insensible nerves in one case of congestive neuralgia, in the other of chronic conjunctivitis, of twenty-five years standing, and of corneitis, with ulcerations of indefinite duration, and the complete immunity from pain in both cases, as soon as that insensibility was removed, and also to the entire removal of inflammatory action by electricity alone, no medication being allowed, either local or general.

These cases taught me that painful inflammatory diseases were most likely confined to individual nerves, and that these nerves were themselves insensible, while producing so much suffering, which view seems paradoxical; but its truth will be tested.

A lady, Mrs. Cook, who has been an invalid for twenty-six years, a subject of rheumatism for twelve years, wheeled about our town by hand for many years, frequently applied at my clinic for relief from her sufferings, called my attention to tender spots near the elbow, which I found to be in the track of certain nerve trunks, viz.: ulnar and radial.

On each spot indicated, I placed a steel electrode, and, pressing it firmly, found it pleasant to the patient, and, taking the course of the nerve, I traced it out through its bifurcations to the tips of the fingers; and while tracing accurately, the steel was not only not painful, but pleasant, always eliciting the gratitude of the patient; but on varying the one-sixteenth of an inch from the nerve track, caused an exclamation of pain. The fingers were much distorted, stiff and partially anchylosed. The right index was so drawn as to over-reach the next two to it, so that the patient used the thumb and the second joint or knuckle of the index in sewing. The anterior aspect of the index was perfectly anæsthetic or insensible to the electric current, but after using the steel electrode thirty minutes I asked her to try to move it. With the effort the finger commenced to shake, and finally moved around into line with its fellows, and the patient uses it now as other women in sewing.

I now find in every case of chronic rheumatism and neuralgia a dead or insensible nerve, at or near the painful spot. It may have tactile sensation, but to the electric current it is indifferent. The first sensations produced by a concentrated current are pleasant, then warmer on to burning, until the instrument must be removed on account of intensity of heat or burning sensation; and the pain is banished at once and the limb or joint is ready to move in any and all natural directions.

I have now treated a sufficient number of cases to satisfy myself of the facts and views as stated, and that all cases of neuralgia or chronic rheumatism or muscular rheumatism may be cured almost instantly if the nerve implicated is accessible or can be found.

---

PRIZE.—A copy of *Lusk's Science and Art of Midwifery*, last edition, was awarded to Dr. W. G. Jameson, of Rusk, for the first club of six subscribers.

